# Antimicrobial, Antioxidant, Sensory Properties, and Emotions Induced for the Consumers of Nutraceutical Beverages Developed from Technological Functionalised Food Industry By-Products

**DOI:** 10.3390/foods9111620

**Published:** 2020-11-06

**Authors:** Egle Zokaityte, Vita Lele, Vytaute Starkute, Paulina Zavistanaviciute, Darius Cernauskas, Dovile Klupsaite, Modestas Ruzauskas, Juste Alisauskaite, Alma Baltrusaitytė, Mantvydas Dapsas, Karolina Siriakovaite, Simonas Trunce, Raquel P. F. Guiné, Pranas Viskelis, Vesta Steibliene, Elena Bartkiene

**Affiliations:** 1 Department of Food Safety and Quality, Faculty of Veterinary, Lithuanian University of Health Sciences, Tilzes Str. 18, LT-47181 Kaunas, Lithuania; egle.zokaityte@lsmuni.lt (E.Z.); vita.lele@lsmuni.lt (V.L.); vytaute.starkute@lsmuni.lt (V.S.); paulina.zavistanaviciute@lsmuni.lt (P.Z.); juste.alisauskaite@stud.lsmu.lt (J.A.); alma.baltrusaityte@stud.lsmu.lt (A.B.); mantvydas.dapsas@stud.lsmu.lt (M.D.); karolina.siriakovaite@stud.lsmu.lt (K.S.); simonas.trunce@stud.lsmu.lt (S.T.); 2 Faculty of Animal Sciences, Institute of Animal Rearing Technologies, Lithuanian University of Health Sciences, Tilzes Str. 18, LT-47181 Kaunas, Lithuania; darius.cernauskas@lsmuni.lt (D.C.); dovile.klupsaite@lsmuni.lt (D.K.); 3 Department of Anatomy and Physiology, Faculty of Veterinary, Lithuanian University of Health Sciences, Tilzes Str. 18, LT-47181 Kaunas, Lithuania; modestas.ruzauskas@lsmuni.lt; 4 Faculty of Veterinary, Institute of Microbiology and Virology, Lithuanian University of Health Sciences, Tilzes Str. 18, LT-47181 Kaunas, Lithuania; 5 CERNAS Research Centre, Polytechnic Institute of Viseu, 3504-510 Viseu, Portugal; raquelguine@esav.ipv.pt; 6 Lithuanian Research Centre for Agriculture and Forestry, Institute of Horticulture, Kauno Str. 30, LT-54333 Babtai, Lithuania; biochem@lsdi.lt; 7 Psychiatry Clinic, Faculty of Medicine, Lithuanian University of Health Sciences, A. Mickeviciaus Str. 9, LT44307 Kaunas, Lithuania; vesta.steibliene@lsmuni.lt

**Keywords:** beverages, milk permeate, wheat bran, fruit/berry by-products, antimicrobial properties, antioxidant properties, overall acceptability, emotions induced for consumers

## Abstract

This study aims to develop nutraceutical beverages containing food processing by-products in their formulation, and determine the opinion of consumers. This is done by testing whether they know that the main ingredients of the product are by-products, performing an overall acceptability test of the developed beverages, and evaluating the emotions induced by the newly developed beverages for consumers. The main ingredients used for the preparation of added-value beverages were fermented milk permeate (containing galactooligosaccharides), extruded and fermented wheat bran (WB) (containing ≥6.0 log_10_ CFU g^−1^ viable antimicrobial properties showing lactic acid bacteria (LAB) strains), and different fruit/berry by-products (FBB) (as a source of compounds showing antioxidant properties). The definition of the quantities of bioactive ingredients was based on the overall acceptability of the prepared beverages, as well as on emotions induced in consumers by the tested beverages. Functional properties of the developed beverages were proofed by the evaluation of their antimicrobial and antioxidant properties, as well as viable LAB count during storage. Desirable changes in extruded and fermented WB were obtained: Fermentation reduced sugar concentration and pH in samples with predominant lactic acid isomer L(+). In addition, the viable LAB count in the substrate was higher than 6.0 log_10_ CFU g^−1^, and no enterobacteria remained. By comparing the overall acceptability of the beverages enriched with WB, the highest overall acceptability was shown for the samples prepared with 10 g of the extruded and fermented WB (7.9 points). FBB showed desirable antimicrobial activity: Shepherd inhibited—2, sea buckthorn—3, blueberries—5, and raspberries—7 pathogens from the 10 tested. Comparing different beverage groups prepared with different types of FBB, in most cases (except sea buckthorn), by increasing FBB content the beverages overall acceptability was increased, and the highest score (on average, 9.5 points) was obtained for the samples prepared with 5.0 and 7.5 g of blueberries FBB. Moreover, a very strong positive correlation (*r* = 0.8525) was found between overall acceptability and emotion “happy” induced in consumers by the prepared beverages enriched with extruded and fermented WB and FBB. By comparing the samples prepared with the addition of WB with samples prepared with WB and FBB, it was observed that most FBB increased total phenolic compounds (TPC) content (on average, by 9.0%), except in the case of samples prepared with sea buckthorn. A very high positive correlation (*r* = 0.9919) was established between TPC and antioxidant activity. Finally, it can be stated that the newly developed nutraceutical beverages were acceptable for consumers, induced positive emotions, and possessed desirable antimicrobial and antioxidant properties, while being prepared in a sustainable and environmentally friendly manner.

## 1. Introduction

According to future prognosis, the global population will increase to 8 billion by 2030 and more than nine billion by 2050, and such population growth will lead to the need for high-quality foods to be assured [1]. However, nowadays, a significant part of the world’s population is suffering from malnutrition [1]. To ensure enough balanced food is available, the food industry must move to become a sustainable industry, in which by-products are very effectively recovered as high-value ingredients and (or) products. However, the food system is highly complex and is driven by many economic, cultural and environmental factors [1]. It should be mentioned that until now, many high-value food industry by-products are used as a low-value feedstock for livestock feeding. At the same time, many people are suffering from biologically active compounds (antioxidants, dietary fibre, etc.) deficiency [1]. As is the case with many food processing industries, by-product recovery can reduce the quantity of wastes that require treatment; however, new technologies and new product formulations should be developed. From another point of view, knowing that the main ingredients of the product are by-products, will the consumer choose it? For this reason, in our study, in addition to the overall acceptability standard test, evaluation of the emotions induced by the newly developed beverages for consumers were measured. Emotion is usually defined as a rapid reaction to a stimulus, which could be a food or drink [2]. The application of emotions evaluation has grown in the last years because it can be used for a prognosis about the emotions induced for consumers by the different food, as well as a choice of food. The emotions induced by food for consumers can be linked to health-related problems [3], and also, can be adapted for commercial product development, to ensure their popularity in the market.

The highest quantity of food-processing by-products is generated by fruit and vegetable, dairy, meat, poultry, olive oil, fermentation, and seafood industries [4]. For this reason, for the development of added-value beverages in this study, milk permeates, wheat bran, and fruit/berries by-products were chosen.

Milk permeate (MP) is a dairy industry by-product obtained during the milk protein concentrate production. The MP, containing a high concentration of lactose, can be used as a stock for galactooligosaccharides (GOS) production [5]. Our previous studies showed that MP fermentation with selected lactic acid bacteria (LAB) strains could lead to additional value formation, by lactose converting to GOS [5]. GOS are desirable compounds in food because consumption of prebiotics is a useful strategy in order to prevent many diseases, and GOS, as a nutraceutical compound, can lead to protective biological functions, e.g., antitumour [6].

Another food industry sectors that generate high quantities of by-products is the wheat processing industry [7,8,9,10,11]. Wheat is the most valuable crop in the world; however, wheat generates very large amounts of by-products (approximate 15% of wheat is not used efficiently), but could be potentially used for the production of value-added products. However, the addition of WB to food formulations usually induces adverse effects on sensory properties of the final product [12]. Extrusion is proposed to increase the acceptability of WB by changing its properties. Extrusion is a combination of thermal and mechanical treatments where the substrate is subjected to high temperature and shear forces for a short time. This process is used to texturise food materials and can have a positive influence on a functional value of WB, e.g., by decreasing antinutritional factors [13,14,15,16]. Moreover, as a high-temperature process, extrusion can lead to reduced microbial contamination, and during the Maillard reaction, the aroma changes to be more acceptable for consumers. Therefore, this process could be used for WB pre-treatment to improve it as a food ingredient, improve sensory properties, as well as to reduce microbial contamination. In addition, fermentation with selected LAB strains can lead to extruded WB having additional value, for example, by providing antimicrobial properties. For this reason, we hypothesise that extruded and fermented WB can be a useful ingredient for additional value beverages development.

Another industry that generates large amounts of by-products is fruit/berries industry. In this study, *Sambucus nigra* L., *Rubus idaeus* L., *Hippophae rhamnoides* L., and *Vaccinium myrtillus* L. by-products were used for the development of additional value beverages. *Sambucus nigra* L., known as elderberry, is a very popular species of the *Adoxaceae* family [17]. Elderberry is a very popular ingredient in many foods and beverage formulations: wine, juice, tea, liqueur, muffins, pancakes, jams and jellies, waffles, batter, etc. [18]. Elderberries are popular in folk and professional medicine, because of high quantities of bioactive compounds possessing desirable characteristics for health improvement [19,20,21,22,23]. Several papers have been published about their antioxidant activities [21,24,25,26].

Red raspberries (*Rubus idaeus* L.) are very popular worldwide and consumed as fresh or processed into a variety of products: confitures, juice, jams, etc. [27]. Due to their phenolic compounds and vitamin C, red raspberries possesses antitumoural, antibacterial, and antioxidant activities [28,29,30,31,32,33,34].

Sea buckthorn (*Hippophae rhamnoides* L.) is an ecologically and economically important plant [35]. Sea buckthorn berries contain a high amount of various hydrophilic and lipophilic compounds, carotenoids, polyphenols, organic, amino and fatty acids, minerals, etc. [36,37,38,39,40]. Sea buckthorn berries are used for high added-value juice and oil production [41]; therefore, the remaining pulp can be used for added-value products development. Blueberry (*Vaccinium myrtillus* L.) species are distributed all over the world [42]. These fruits are usually consumed in the fresh form, however, due to their short shelf life, they are used for jams, juices, wines, or liqueurs production [43]. These berries are conventionally used in medicine [44], because of their high content of phenolics and carotenoids, as well as vitamins. The European blueberry is an economically valuable wild berry, well-known for its richness of antioxidants (anthocyanins) [42,43]. Blueberries are used for various food and beverage preparation, including juice and wine, and these processes generate valuable by-products, which can be used for further added-value product development.

The aim of this study was to develop additional value beverages in a sustainable manner by using a formulation of food processing by-products. The main ingredients for additional value beverage preparation were fermented milk permeate (containing GOS), extruded and fermented wheat bran (WB) (containing ≥6.0 log_10_ CFU g^−1^ viable antimicrobial properties showing LAB), and different fruit/berry by-products (as a source of antioxidant properties showing compounds). The main selection of the quantities of bioactive ingredients was based on the prepared beverages ‘overall acceptability, as well as on emotions induced by the tested beverages for consumers. Functional properties of the developed beverages were proofed by the evaluation of their antimicrobial and antioxidant properties, as well as viable LAB count in the developed drinks during the storage.

## 2. Materials and Methods

**Figure 1:**
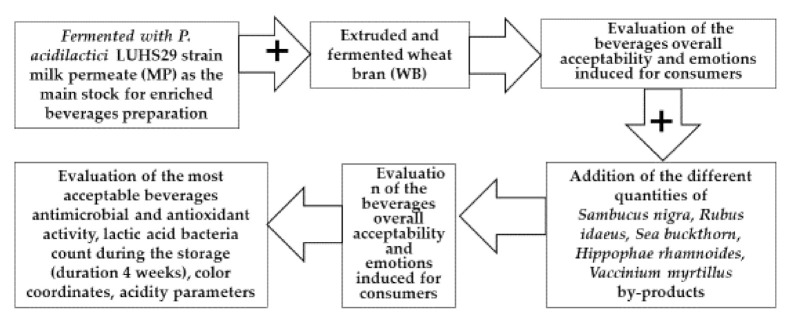
The experimental scheme.

The whole experiment scheme is shown in Figure 1.

### 2.1. Characteristics of Fermented Milk Permeate used for Beverages Preparation

Milk permeate (MP) was obtained from the Agricultural cooperative “Pienas LT”, Biruliskes, Lithuania. Our previous studies showed that the highest concentration of galactooligosaccharides (GOS) and the most effective antimicrobial properties of MP could be obtained when *P. acidilactici* LUHS29 strain was used for MP fermentation [5]. Characteristics of the fermented MP, used in this study for enriched beverages preparation are shown in Table 1 (acidity parameters, LAB count, GOS concentration, overall acceptability and emotions induced for consumers) and Table 2 (antimicrobial properties).

### 2.2. Wheat Bran, Used for Beverages Enrichment, by Using it for Pre-Treatment Extrusion and Fermentation Processes

Wheat bran was obtained from the SME “Ustukiu malunas” (Pasvalys, Lithuania). Wheat bran samples (WB) were extruded at 130 °C, speed of the screw—25 rpm and fermented with *L. uvarum* LUHS245 strain. The LUHS245 strain, before the experiment, was stored at −80 °C in a Microbank system (Pro-Lab Diagnostics, Birkenhead, Wirral, UK) and grown in de Man, Rogosa and Sharpe (MRS) broth (CM 0359, Oxoid, Basingstoke, Hampshire, UK) at 30 °C for 48 h prior to use.

The following parameters for WB were established: pH, total titratable acidity (TTA), L(+) and D(-) lactic acid bacteria concentration, LAB, mould/yeast (M/Y), total bacteria (TBC), and total enterobacteria (TEC) counts; sugars concentration (fructose, glucose, sucrose, maltose); amino acids and biogenic amines concentration. Non-extruded and non-fermented WB samples were used as control.

#### Wheat Bran Analysis Methods

The pH was measured using a pH electrode (PP-15; Sartorius, Goettingen, Germany). The total titratable acidity (TTA) was evaluated for a 10 g sample of sample mixed with 90 mL of water, and the results were expressed in mL of 0.1 mol L^−1^ NaOH solution required to achieve a pH value of 8.2. For L(+) and D(−) lactic acid isomers concentration evaluation, a specific Megazyme assay Kit (Megazyme Int., Bray, Ireland) was used. The determination of LAB, total bacteria (TBC), enterobacteria (TEC), and mould/yeast (M/Y) counts in samples was performed according to Bartkiene et al. [45].

To determine the sugar concentration, 2–3 g of sample was diluted with ~70 ml of distilled/deionised water, heated to 60 °C in a water bath for 15 min, clarified with 2.5 ml Carrez I (85 mM K_4_[Fe(CN)_6_] × 3H_2_O) and 2.5 ml Carrez II (250 mM ZnSO_4_ × 7H_2_O) solutions, and made up to 100 ml with distilled/deionised water. After 15 min, the samples were filtered through a filter paper and a 0.22 μm nylon syringe filter before analysis. A standard solution of a sugar’s mixture was prepared by dissolving 0.2 g each of fructose (Hamburg, Germany), glucose (Sigma-Aldrich, Hamburg Germany), sucrose (Sigma-Aldrich, Hamburg Germany) and maltose (Sigma-Aldrich, Hamburg, Germany) in 100 mL of distilled/deionised water. A 2 mg mL^−1^ standard solution of sugars mixture was prepared following dilution with distilled/deionised water. Chromatographic conditions were as follows: The eluent was a mixture of 75 parts by volume of acetonitrile and 25 parts by volume water, the flow rate was 1.2 mL/min, 20 μL was injected. The YMC-Pack Polyamine II 250 × 4.6 mm, 5 μm (YMC Co., Ltd., Tokyo, Japan) column was used. The column temperature was set at 28 °C. Detection was performed using an Evaporative Light Scattering Detector ELSDLTII (Shimadzu Corp., Kyoto, Japan).

Free amino acids (FAA) were extracted using 0.1 M HCl. The extracts were analysed by gas chromatography with flame ionisation detection after an ion-exchange solid-phase extraction and chloroformate derivatisation using EZ:faast technology (Phenomenex) as described by Bartkiene et al. [46].

The extraction and determination of biogenic amines (BA) in wheat samples followed the procedures developed by Ben-Gigirey et al. [47] with some modifications, as described by Bartkiene et al. [48].

### 2.3. Fruits/Berries By-Products used for Milk Permeate Beverages Preparation

Four different fruit/berry by-products types (Shepherd/*Sambucus nigra*, Raspberries/*Rubus idaeus*, Sea buckthorns/*Hippophae rhamnoides*, Blueberries/*Vaccinium myrtillus)* were obtained from the Institute of Horticulture, Lithuanian Research Centre for Agriculture and Forestry (Babtai, Kaunas distr., Lithuania) in 2020. These by-products were vacuum dried in a vacuum dryer XF020 (France-Etuves, Chelles, France) at 45 ± 2.0 °C and a pressure of 6 × 10^−3^ mPa. The antimicrobial and antifungal properties for the selected fruit/berry by-products were evaluated.

#### Antimicrobial Properties of the Fruit/Berry By-Products Evaluation

The antimicrobial activity of fruit/berry by-products was evaluated against a variety of pathogenic and opportunistic bacterial strains (*Salmonella enterica Infantis* LT 101, *Staphylococcus aureus* LT 102, *E. coli* (hemolytic) LT 103, *Bacillus pseudomycoides* LT 104, *Aeromonas veronii* LT 105, *Cronobacter sakazakii* LT 106, *Hafnia alvei* LT 107, *Enterococcus durans* LT 108, *Kluyvera cryocrescens* LT 109, *Acinetobacter johnsonii* LT 110). The pathogenic and opportunistic bacterial strains used were obtained from the Lithuanian University of Health Sciences’ (Kaunas, Lithuania) collection. The antimicrobial activity of the fruit/berry by-products was assessed by measuring the diameter of inhibition zones (DIZ, mm) in agarwell diffusion assays. Accordingly, a 0.5 McFarland unit density suspension of each pathogenic bacteria strain was inoculated onto the surface of cooled Mueller–Hinton agar (Oxoid, Basingstoke, UK) using sterile cotton swabs. Wells of 6 mm in diameter were punched in the agar and filled with the tested by-product. Before the experiment, the fruit/berry by-products were diluted with a sterile physiological solution (1 g of the by-product diluted with 2 mL of the physiological solution). The average DIZ was calculated from triplicate experiments.

### 2.4. Selection of the Optimal Quantities of Technologically Functionalised Wheat Bran for Milk Permeate Beverages Enrichment

The different quantities (2.5, 5.0, 7.5, and 10 g) of WB were added to the fermented MP samples (50 mL), and the most acceptable samples for the further enrichment with fruit/berry by-products were selected. In addition, emotions induced for consumers by the prepared beverages enriched with extruded and fermented WB were evaluated. Description of the overall acceptability and emotions induced for consumers by the prepared beverages are described below in Section 2.3.

#### Overall Acceptability and Emotions Induced for Consumers by the Prepared Beverage Enriched with Wheat Bran Beverages Evaluation

The overall acceptability of the beverages was established by 50 judges, according to International Standards Organisation method 8586-1 [49], using a 10-point scale ranging from 0 (“extremely dislike”) to 10 (“extremely like”). Similarly, the prepared beverages were tested by applying FaceReader 6.0 software (Noldus Information Technology, Wageningen, The Netherlands), scaling nine emotion patterns (neutral, happy, sad, angry, surprised, scared, disgusted, contempt, and valence) according to Bartkiene et al. [50]. In the obvious measurement experiment, subjects were asked to rate the beverage samples during and after consumption with an intentional facial expression, which was recorded and then characterised by FaceReader 6.0. The participants were asked to taste the whole presented sample at once, take 15 s to reflect on the taste impressions, then give a signal with a hand and visualise the taste experience of the sample with a facial expression best representing their liking of the sample. The whole procedure was filmed using high-resolution Microsoft LifeCam Studio webcam mounted on a laptop facing the participants, and Media Recorder (Noldus Information Technology, Wageningen, The Netherlands) software. Special care was taken to ensure good illumination of participant’s faces. The recordings, using a resolution of 1280 × 720 at 30 frames per second, were saved as AVI files and analysed frame by frame with FaceReader 6 software, scaling the nine basic emotion patterns (neutral, happy, sad, angry, surprised, scared, disgusted, contempt and valence) to 1 (maximum intensity of the fitted model). In addition, the FaceReader also analysed the valence, which indicates whether the person’s emotional status is positive or negative. ‘Happy’ is the only positive emotion, while ‘Sad’, ‘Angry’, ‘Scared’, and ‘Disgusted’ are considered to be negative emotions. ‘Surprised’ can be either positive or negative. The valence is calculated as the intensity of ‘Happy’ minus the intensity of the negative emotion with the highest intensity. Valence scores ranged from -1 to 1. For each sample, the section of intentional facial expression (from the exact point at which the subject had finished raising their hand to give the signal until the subject started lowering their hand again) was extracted and used for statistical analysis.

### 2.5. Selection of the Optimal Quantities of Fruits/Berries By-Products for Milk Permeate Beverages Enrichment

The different quantities (2.5, 5.0, 7.5, 10 g) of fruit/berry by-products (Shepherd/*Sambucus nigra,* Raspberries/*Rubus idaeus*, Sea buckthorns/*Hippophae rhamnoides*, Blueberries/*Vaccinium myrtillus)* were tested. In addition to the optimal quantity of WB, the optimal quantity of the tested fruit/berry by-products was selected. First of all, the optimal quality was selected by the evaluation of overall acceptability and emotions induced for consumers by the prepared enriched with WB and fruit/berry by-products beverages (methods described in Section 2.3.

After an optimal (according to overall acceptability and induced emotions) fruit/berry by-products content selection, the most acceptable samples were analysed further, by evaluating prepared enriched beverages antimicrobial properties, LAB count during the storage, colour coordinates, and acidity parameters. Description of the above-mentioned methods is given in.

#### Antimicrobial Activity of the Prepared Beverages Enriched with Wheat Bran and Fruits/Berries By-Products

Antimicrobial activity of the prepared beverages enriched with extruded and fermented wheat bran and fruit/berry against a variety of pathogenic and opportunistic bacterial strains (*Salmonella enterica Infantis* LT 101, *Staphylococcus aureus* LT 102, *E. coli* (hemolytic) LT 103, *Bacillus pseudomycoi*des LT 104, *Aeromonas veronii* LT 105, *Cronobacter sakazakii* LT 106, *Hafnia alvei* LT 107, *Enterococcus durans* LT 108, *Kluyvera cryocrescens* LT 109, *Acinetobacter johnsonii* LT 110) was evaluated. The used pathogenic and opportunistic bacterial strains were attained from the Lithuanian University of Health Sciences (Kaunas, Lithuania) collection. Antimicrobial activity was assessed by measuring the diameters of inhibition zones (DIZ, mm) in agar well diffusion assays. For this purpose, 0.5 McFarland unit density suspension of each pathogenic bacteria strain was inoculated onto the surface of cooled Mueller–Hinton agar (Oxoid, UK) using sterile cotton swabs. Wells of 6 mm in diameter were punched in the agar and filled with 50 µL of the prepared beverages samples. The average DIZ was calculated from triplicate experiments.

Acidity parameters (pH and TTA) of the prepared beverages enriched with extruded and fermented wheat bran and fruit/berries were evaluated immediately after preparing the beverages. The pH value of beverages was measured and recorded using a pH electrode (PP—15, Sartorius, Goettingen, Germany). The total titratable acidity (TTA) was determined of a 10 mL sample homogenised with 90 mL distilled water and expressed as the amount (mL) of 0.1 mol L^−1^ NaOH to obtain a pH value of 8.2.

The colour coordinates (L*, a*, b*) were assessed using a CIELAB system (Chromameter CR-400, Konica Minolta, Tokyo, Japan).

For the evaluation of LAB count, 10 mL of the beverage were homogenised with 90 mL of saline (9 g L^−1^ NaCl solution). Serial dilutions of 10^−4^ to 10^−8^ with saline were used for sample preparation. Sterile MRS agar (CM0361, Oxoid) of 5 mm thickness was used for bacterial growth on Petri dishes. The dishes were separately seeded with the sample suspension using surface sowing and were incubated under anaerobic conditions at 30 °C for 72 h. All results were expressed in log_10_ CFU mL^−1^ (colony forming units per mL of the sample) as the mean of three determinations. To determine the viability of LAB during four weeks of storage at +4 °C.

### 2.6. Statistical Analysis

The results were expressed as the mean ± standard deviation (SD). All analyses were performed at least in triplicate. Results were analysed using statistical package SPSS for Windows V15.0 (SPSS Inc., Chicago, IL, USA, 2007). The significance of differences between the samples was evaluated using Tukey range tests at a 5% level. A linear Pearson’s correlation was used to quantify the strength of the relationship between the variables. The correlation coefficients were calculated using the statistical package SPSS. The results were recognised as statistically significant at *p* ≤ 0.05.

## 3. Results

### 3.1. Parameters of the Extruded Wheat Bran

Acidity (pH, total titratable acidity (TTA), and lactic acid isomers concentration) and microbiological parameters (lactic acid bacteria (LAB), mould/yeast (M/Y), total bacteria (TBC), and total enterobacteria (TEC) count) of the extruded and fermented WB are shown in Table 3. In comparing fermented and non-fermented WB samples, after 24 h of fermentation, the samples‘ pH was reduced by 28.9%, and TTA increased by 94.3%, in comparison with non-fermented extruded samples. L(+)/D(−) ratio in fermented WB samples was 1.35, with predominant L(+) lactic acid. Lactic acid bacteria (LAB) count in fermented samples was, on average, 8.79 log_10_ CFU g^−1^, however, significant reduction of M/Y count in fermented samples was not observed, compared with non-fermented ones. Enterobacteria did not remain in the fermented samples, however, extrusion was not a significant factor for TEC. Moreover, fermentation reduced sugar concentration in WB samples, and fructose, sucrose, and maltose did not remain after fermentation.

Technological microorganisms, such as LAB, produce a variety of organic acids in the substrate and lower the pH to levels that are inhibitory to many pathogenic and opportunistic microorganisms [51]. The increases of TTA and the reduction of pH improves food safety parameters as well. The levels of pH and TTA in the substrate are influenced by many factors, including processing methods and product properties.

The major metabolite of LAB is D(−) and/or L(+) lactic acid [52]. However, the studies revealed the microbiota metabolism of D(−) and L(+) lactic acid in fermented products are scarce. Data on the ratio of lactic acid isomers was published for sauerkraut and cheese [53,54]. Different LAB showed different production ratios of D and L lactic acid [52,55]. The accumulation of D(−) lactic acid may cause D-lactic acidosis in mammals [56,57]. For this reason, researchers aimed to reduce the accumulation of D(−) isomers in fermented foods during the fermentation.

The contamination of foods during the various processes of the production chain is always the point of concern; this is especially important for the outer layer of cereals, which are contaminated from the field. Moreover, the occurrence of some fungal species can be a signal of mycotoxin contamination [58,59,60]. Different methods, including thermal and non-thermal, are used to decrease the bacterial, as well as fungal contamination of the cereal-based products [58,61]. The most environmentally-friendly and efficient methods for cereal decontamination are fermentation processes. In addition, fermentation leads to some positive nutritional and sensory characteristics of the products [61]. Moreover, during the extrusion process, which includes a combination of high temperature and high pressure, toxin, as well as non-desirable microorganisms reduction in food can be observed [62].

It was published that WB contamination (TBC) before fermentation was 5 log_10_ CFU g^−1^. For this reason, to ensure the stability of the fermentation process with *L. rhamnosus* 1473 strain, sterilisation step was included [63].

Finally, desirable changes in the fermented substrate were obtained: Fermentation reduced the sugar concentration and pH in the samples with predominant lactic acid isomer L(+), and also, the viable LAB count in the substrate was higher than 6.0 log_10_ CFU g^−1^ and enterobacteria did not remain.

The amino acids and biogenic amines (Bas) concentration in nontreated and extruded, as well as extruded and fermented cereal by-products, are shown in Table 4. Most of the analysed amino acids concentration after extrusion, as well as extrusion and fermentation in WB samples, remain similar as before treatment, however, some changes were established in glutamine, cysteine, tryptophan, phenylalanine and isoleucine content. Glutamine concentration in extruded and extruded/fermented WB samples was, on average, by 17.1% lower, compared with nontreated WB. An opposite tendency with cysteine concentration was found, and in comparison, nontreated and fermented/extruded WB samples, were on average, 15.0% higher in concentration in treated samples. However, tryptophan, phenylalanine, and isoleucine concentrations were reduced after extrusion and fermentation, on average, by 19.4, 21.4 and 20.0%, respectively. In opposite, lysine concentration in extruded and fermented samples was significantly higher (on average, by 23.5%).

Despite the fact that WB fermentation is a very popular process in the food and feed industry, however published studies are very scarce [64,65,66], and it is worthy of note that about an extrusion and fermentation combination for WB treatment was never reported before [63].

Overall, WB is the main by-product of the wheat milling industry, containing more than 15% protein [67]. It was published that the proteins can be derived from WB [68]. Despite the fact that endosperm biological value is higher in comparison with bran, however, WB proteins have a more favourable amino acid composition compared to endosperm proteins [69]. However, these proteins are located within cell wall polysaccharides, and for this reason, its digestion is pure [70]. Compared to endosperm, WB proteins contain a higher amount of lysine, arginine, and glycine [71]. The most dominant amino acids in WB are glutamic and aspartic acids, leucine, alanine, proline, arginine, and glycine [70]. However, such a high content of protein in WB can lead to biogenic amines (BA) formation, especially, during the fermentation processes [72].

In comparing Bas concentration in WB, phenylethylamine, tyramine, and spermidine were not found in the tested WB samples. However, putrescine and spermine concentration in WB was increased after extrusion and extrusion/fermentation processes (in extruded samples by 33.6%, in extruded and fermented WB by 36.1% higher, in comparison with nontreated WB). Cadaverine and histamine were found just in the nontreated WB (on average, 41.33 and 63.64 mg kg^−1^, respectively). In opposite to putrescine, spermidine concentration after both treatments in WB was reduced (in extruded samples by 70.9%, in extruded and fermented WB by 70.1% lower, in compare with nontreated WB).

Bas are non-volatile nitrogenous bases with an aliphatic, aromatic structure formed by the decarboxylation of free amino acids [73,74,75]. Depending on chemical structures, Bas are aromatic amines (histamine, tyramine, β-phenylethylamine, tryptamine and serotonin), aliphatic diamines (putrescine and cadaverine), and aliphatic polyamines (agmatine, spermidine, and spermine). It has been published that BA antioxidant properties are stronger than those of some antioxidant vitamins [76]. BA concentrations varied widely within food types [74,77,78,79], and they can be influenced by stock origin, processing, storage technology etc. [74,77,80]. It should be pointed out that the consumption of foods high BA concentrations may be deleterious to human health; for this reason, it is very important to estimate concentrations of BA in foods [74,77,80]. Bas are stable compounds [74], however, it has been published that the milling process influences BA distribution in different cereal fractions [81]. It has been reported that whole-grain wheat contains greater amounts of polyamines in comparison with bread [82]. Confirmed results on the BA content in different fractions of cereal grains are limited, however, it is known that histamine, putrescine, cadaverine, tyramine, spermidine and spermine are responsible for toxicological effects of foods [81]. As low molecular weight compounds, after ingestion, they rapidly appear in the blood and various organs and are can inducing several digestive, circulatory and respiratory symptoms [74].

It has been published that BA concentration in durum wheat cultivars is considerable, but not so high as in fish, meat, cheese, fermented vegetables, soy products, and alcoholic beverages, etc. [73,74,79]. Finally, despite that the cereals have low BA content in comparison to the other foods, together with other high-BA foods, they can enhance allergic reactions [73,74]. To prevent the non-desirable effects of food, it is very important to control BA in a wide range of products [74].

### 3.2. Antimicrobial Properties of the Fruits/Berries by-Products

The antimicrobial properties of the fruit/berry by-products are shown in Table 5. Selected for this experiment, fruit/berry by-products (shepherd, raspberries, sea buckthorn, blueberries) did not show inhibition properties against *Salmonella enterica Infantis* and *Kluyvera cryocrescens*. Only raspberry by-products inhibited *E. coli (hemolytic)*, *Aeromonas veronii* and *Cronobacter sakazakii*, with the diameter of inhibition zones (DIZ) being on average 12.9 mm. All the tested fruit/berry by-products inhibited *Enterococcus durans*, and the highest DIZ of the sea buckthorn against this pathogen was found (15.4 mm). Raspberries, sea buckthorn, and blueberries by-products showed antimicrobial properties against *Bacillus pseudomycoides* and *Acinetobacter johnsonii*, and the highest DIZ against both pathogens by raspberries by-products was found (on average, 15.4 mm). *Staphylococcus aureus* was inhibited by shepherd and blueberries by-products (DIZ, 13.3 and 9.2 mm, respectively). *Hafnia alvei* was inhibited by raspberries and blueberries by-products (DIZ 10.5 and 10.7 mm, respectively).

*Sambucus nigra* L. is well known because of its natural compounds, which reduces oxidative stress-induced diseases. Shepherd contains various organic acids, flavanol glycosides and anthocyanins [83,84]. The anthocyanins present in shepherd showed protective effects against influenza A and B virus and Helicobacter pylori infections [85,86,87], and work has been published about shepherd’s antifungal, antitumour [88,89,90,91,92,93,94] and antimicrobial properties [95]. The main compounds responsible for shepherd’s antimicrobial properties are polyphenols; extracts of shepherd possess antibacterial activity against E. coli and Pseudomonas pudita, however inhibition of *Bacillus cereus* and *Staphylococcus aureus* was not established in the literature.

Raspberry juice possesses antimicrobial and antifungal activity against *Staphylococcus aureus*, *Escherichia coli*, *Proteus vulgaris*, *Pseudomonas aeruginosa*, *Bacillus subtilis*, and *Candida albicans*. Antimicrobial activity of raspberries is explained by the presents of ellagitannins, whose content and composition may vary depending on the variety and geographical location, however, it has been published that raspberry extracts inhibited both Gram-positive and Gram-negative bacteria [96]. Sea buckthorn berries are rich in carotenoids, tocopherols, fatty acids, antioxidants, flavonoids, ascorbic and organic acids [97]. The main identified components are ascorbic acid, carotenoids and various phenolics, including proanthocyanins, gallic acid, ursolic acid, caffeic acid, cumaric acid, ferulic acid, catechin and epicatechin derivatives, quercetin, kaempferol, and isorhamnetin glycoside derivatives [98,99,100,101], which can be associated with pathogenic inhibition properties.

Blueberry fruits have revealed antimicrobial properties against *Citrobacter freundii* and *Enterococcus faecalis* [102]. It was published that blueberry leaves inhibited *S. aureus*, and this result can be related to high phenolic compounds content, which attacks an important number of bacteria, with the antimicrobial capacity depending on the interactions between polyphenols and bacterial cell surface [103,104]. *R. equi* was the most sensitive strain towards blueberry extracts, whereas *E. faecalis* Gram-positive strain was the most resistant one [105].

Fruit/berry by-products showed desirable antimicrobial activity: Shepherd inhibited 2, sea buckthorn—3, blueberries—5, and raspberries—7 pathogens from the 10 tested. Finally, in this study, shepherd by-products, and the obtained results showed that the tested by-products were very promising antimicrobial ingredients for nutraceuticals, pharmaceuticals, and food formulations.

### 3.3. Overall Acceptability and Emotions Induced for Consumers by the Prepared Enriched with Wheat Bran Beverages

The overall acceptability and emotions induced for consumers by the prepared beverages enriched with extruded and fermented wheat bran (WB) are shown in Table 6. Comparing the overall acceptability of the prepared beverages enriched with the different treated WB, the highest overall acceptability of the samples, prepared with 10 g of the extruded and fermented WB, is shown (7.9 points).

Today, scientific interest focuses not just on food’s nutritional and functional value, but also on food-induced emotional responses, because, emotions are closely related to consumers’ food choices [106,107]. It has been published that the disliking of unknown and/or non-traditional foods is strongly related to negative emotions [108]. It is also known that positive emotions, such as joy, happiness, and satisfaction, have a significant positive correlation with food’s sensory properties [109]. According to Dalenberg et al. [110], emotional responses better-characterised food choices in comparison with liking. However, in comparison with other affective feelings, emotions were characterised by high intensity, rapid change, and were short-lasting [111].

In this study, between overall acceptability and the emotion “disgusted” was induced for consumers by the prepared beverages enriched with extruded and fermented WB, but very weak positive correlations were found (*r* = 0.1467), as well as weak positive correlations between overall acceptability and emotions “neutral”, “happy”, “sad”, and “angry” were found (*r* = 0.2430, *r* = 0.2105, *r* = 0.2705, and *r* = 0.2439, respectively). The strongest (positive moderate) correlation between the overall acceptability and emotion “scared” was found (*r* = 0.5295). According to the results obtained, for the further experiment, samples prepared with 10 g of the extruded and fermented WB was chosen, as they showed the highest overall acceptability.

### 3.4. Overall Acceptability and Emotions Induced for Consumers by the Prepared Beverages Enriched with Wheat Bran and Fruits/Berries By-Product Beverages

Overall acceptability and emotions induced for consumers by the prepared beverages enriched with extruded and fermented WB and fruit/berry by-products are shown in Table 7. In comparison, different beverage groups prepared with different types of berries, in most of the cases (except sea buckthorn), by increasing the berries’ content, the beverages’ overall acceptability was increased, and the highest overall acceptability of the samples, prepared with 5.0 and 7.5 g of blueberry by-products was found (on average, 9.5 points).

Evaluation of the induced emotions by brands, packaging, etc. is generally performed to obtain information about product sales, brand loyalty, and consumer satisfaction [112]. However, the study of emotions induced by unpackaged foods and beverages in response to their sensory properties is more recent and very important for the development of product innovations [113,114]. It is suggested that the sensory properties of a product may correlate with emotions, and for this reason, a greater understanding of the relationship between sensory characteristics and emotions has become very important [115,116].

In this study, between overall acceptability and the emotion “happy” induced for consumers by the prepared beverages enriched with extruded and fermented WB and berries, there was a very strong positive correlations were found (*r* = 0.8525), as well as a strong negative correlation between overall acceptability and emotion “angry” was found (*r* = −0.6842). Moderate negative correlations between the overall acceptability and emotions “disgusted” and “contempt” were found (*r* = −0.4134 and *r* = −0.4134, respectively). Between overall acceptability and emotions “neutral” and “sad” very weak positive correlations were found (*r* = 0.1136 and *r* = 0.1973, respectively). According to overall acceptability results, for the further experiments, samples prepared with 20 g 50 mL^−1^ of WB and with the addition of 5.0 g 50 mL^−1^ of sea buckthorn and 7.5 g 50 mL^−1^ of shepherd, raspberries and blueberries were selected.

In the last decade, evaluation of emotions has been widely applied by the beverage industry in the product development cycle, for product improvement and optimisation, and changes in the formulation [117,118]. However, the literature in this area of application is scarce, since most manufacturers use this information internally to achieve a technical advantage against other competitors in the market [117]. Thomson et al. [119] published that specific sensory characteristics are associated with emotional conceptualisations in unbranded samples of dark chocolate, including associations of “cocoa” with “powerful” and “energetic”, “bitter” with “confident”, “adventurous” and “masculine”, and “creamy” and “sweet” with “fun”, “comforting” and “easy-going”. However, Thomson et al. did not compare hedonic and emotional responses; for this reason, it is not possible to determine sensory-emotion linkages. However, a correlation between acceptability and emotional associations in food and beverages was reported [114,120,121,122,123,124].

In this study, also, a very strong positive correlation was found between overall acceptability and the emotion “happy”, however, it should be mentioned that the beverages without fruit/berry by-products showed lower correlations between overall acceptability and induced emotions. It could be that more intensive sensory properties induced by the addition of fruit/berry by-products, induced stronger emotions for consumers, which were fixed, and in this study, by fruit/berry by-products induced emotions were positive.

### 3.5. Antimicrobial Activity of the Prepared Beverages Enriched with Wheat Bran and Fruits/Berries By-Products

The DIZ of the prepared beverages against pathogenic and opportunistic strains are shown in Table 8. All of the prepared beverages showed inhibition properties against *Salmonella enterica Infantis* and *Staphylococcus aureus*, however, all of the prepared beverages did not inhibit *Kluyvera cryocrescens.* Beverages, prepared with extruded and fermented WB, but without berries/fruits by-products inhibited 2 out of 10 tested pathogenic and opportunistic strains, however, beverages prepared with shepherd and sea buckthorn inhibited 9 out of 10, as well as beverages prepared with raspberry and blueberry by-products, which inhibited 8 out of 10 tested pathogenic and opportunistic strains. The highest DIZ of beverages prepared with shepherd against *E. coli* (hemolytic) and *Enterococcus durans* were found (13.4 and 12.3 mm, respectively), the highest DIZ of beverages prepared with raspberry by-products against *Bacillus pseudomycoides*, *Enterococcus durans*, and *Acinetobacter johnsonii* (DIZ, on average, 13.8 mm), the highest DIZ of beverages prepared with sea buckthorn by-products against *Enterococcus durans* and *Acinetobacter johnsonii* (DIZ, on average, 13.9 mm), and the highest DIZ of beverages prepared with blueberry by-products against *E. coli* (hemolytic) and *Enterococcus durans* (DIZ, on average, 14.8 mm).

Finally, in all the cases, berries/fruits by-products increase beverages’ antimicrobial properties, in comparison with beverages prepared just with extruded and fermented WB, and these results can be related to berries/fruits’ bioactive compounds and antimicrobial properties, which are described above (Section 3.2). Moreover, during fermentation, LAB excreted a broad spectrum of antimicrobial compounds (organic acids, low molecular weight peptides, hydrogen peroxide, etc.) that inhibits the growth of pathogenic and opportunistic strains [125]. The antimicrobial activity of LAB against a variety of pathogenic and opportunistic strains was determined in several studies [126,127,128,129,130,131]. In the developed beverages, both antimicrobial ingredients: Viable LAB and fruit/berry by-products showed a symbiotic effect on pathogens inhibition.

### 3.6. LAB Count during the Storage, Colour Coordinates, and Acidity Parameters

The viable LAB count in prepared beverages during the four weeks of storage at +4 °C temperature is shown in Table 9. The LAB count after 24 h in beverages was, on average, 8.17 log_10_ CFU mL^−1^, and after one and two weeks of storage, significant changes in the LAB counts were not found. However, after three weeks of storage, LAB count was reduced in the fermented milk permeate (without WB and berries/fruits by-products addition) samples (on average, by 10.3%). After four weeks of storage, higher than 6.0 log_10_ CFU mL^−1^ remain in two beverage groups: Beverages prepared with extruded and fermented WB (on average, 7.20 log_10_ CFU mL^−1^) and in beverages prepared with extruded and fermented WB and 7.5 g 50 mL^−1^ of shepherd (on average, 6.93 log_10_ CFU mL^−1^). Finally, three weeks storage time for beverages can be recommended, because during this time the viable LAB count in beverages remained higher than 6.0 log_10_ CFU mL^−1^. In addition, the beverages prepared with extruded and fermented WB, and beverages prepared with extruded and fermented WB and 7.5 g 50 mL^−1^ of shepherd had their functional properties retained for longer, and for the above-mentioned beverages, four weeks storage time can be recommended.

Colour coordinates, acidity and antioxidant parameters of the prepared beverages are shown in Table 10.

The highest lightness (L*) coordinates of the beverages prepared with extruded and fermented WB were established (39.1 NBS), the lowest L* (by 49.6% lower) of the beverages, prepared with WB and shepherd addition were found. The addition of raspberries increases redness (a*) of beverages, and in comparison with beverages groups with and without berries, beverages with raspberries had a* coordinates that were by 88.0 and 63.5% higher, respectively. The highest yellowness (b*) of the beverages prepared with the sea buckthorn was found (15.5 NBS), and in comparison with other beverages, this group showed, on average, 7.7 times higher b* coordinates. Colour characteristics are one from the main sensory properties, which have a strong relationship with consumers’ acceptance and purchasing decisions regarding a product [132]. In addition, colour is a product quality indicator and influences the perception of taste, safety, as well as nutritional value [133].

Moderate positive correlations between overall acceptability and L*, between emotion “sad” and a*, and between the emotion “disgusted” and L* were found (Table 11). As well as moderate negative correlations between emotion “angry” and a*, it was also established between the emotion “surprised” and a* and b* colour coordinates. In addition, there were strong positive correlations between the emotion “neutral” and L*, between the emotion “happy” and a*, between the emotion “contempt” and a*, and between “valence” and a* and b* coordinates. The strong negative correlation between emotion “happy” and L* was established, as well as a very strong positive correlation between the emotion “sad” and b* and between emotion “contempt” and b*.

In a comparison of the pH of the beverages prepared with functional additives, the lowest pH of the samples prepared with raspberries and blueberries was found to be 4.17 and 4.20, respectively, but it should be mentioned, that all the samples prepared with additives showed a higher pH than that fermented milk permeate (pH 3.91) without WB and/or fruits/berries. A very strong negative correlation was found between pH and TTA of the samples (*r* = –0.94524). In comparison, with total phenolic compound (TPC) content in samples, the highest TPC content in beverages prepared with the addition of raspberries was established (141.7 mg 100 g^−1^ d.m.). The lowest TPC content in non-fermented milk permeate was found (68.2 mg 100 g^−1^ d.m.), however, fermentation increased TPC in milk permeate samples, on average, by 34.9%, compared with samples prepared with extruded and fermented WB with fermented milk permeate without additives, where WB addition increased TPC content, on average, by 15.8%. When comparing the samples group prepared with the addition of extruded and fermented WB with samples prepared with WB and fruit/berry by-products, most fruits/berry by-products increased TPC content in the beverages (on average, by 9.0%), except samples prepared with sea buckthorn, in which TPC remained similar as before the addition of fruit/berry by-products. A very high positive correlation was established Between TPC and antioxidant activity of the samples (*r* = 0.9919).

The development of plant-derived nutraceutical beverages with antioxidant properties has been the intensively studied in recent years [134]. In this study, the main antioxidant properties in the developed beverages’ ingredients were fruit/berry by-products, however, it should be mentioned that the LAB also excreted antioxidant property possessing compounds.

The modulation of the intestinal redox environment using viable bacteria possessing antioxidant properties has also been noted [135].

The health benefits of products containing desirable bioactive compounds have been previously published. Antioxidant characteristics of plants can be related to several anti-oxidative mechanisms of the chemical composition of plant tissues, as well as by micro- and macrocompounds interactions, including synergistic or opposite mechanisms of action [136].

The main compounds, which lead to shepherd antioxidant activity, are anthocyanins and flavanols [137]. Shepherd phenolics are depended on plant genetic differences, environmental conditions, degree of maturity, etc., and these factors are very important for industry because chemical composition is related to antioxidant capacity [138]. Raspberries are a good source of bioactive phytochemicals, especially phenolics, in which the general structure contains an aromatic ring with one or more hydroxyl groups, and these compounds are highly associated with antioxidant capacity [139]. The antioxidant capacity of phenolics is based on the ability of the phenolic ring to stabilise and delocalise unpaired electrons [140].

TPC in raspberries varied between 142 and 758 mg gallic acid equivalents (GAE) 100 g^−1^ fw [29]. The concentration of TPC in plants can be induced by many factors, including species, cultivar, ripening stage, soil, and climate [141,142], producing differences in the TPC found among the different studied species [96]. Moreover, in vitro antioxidant activity of the fruit/berries can be related to the high content of ascorbic acid [143,144,145]. It was reported that Sea buckthorns are rich in phenolics and flavonoids with potential antioxidant and antiproliferative activities and can be recommended in antioxidant and anticancer dietary supplement synthesis and utilisation in the food industry. Furthermore, it was published about blueberry antioxidant activity [102]. The effect of blueberry juice phytochemicals occurs through redox- and non-redox-regulated mechanisms and protects from oxidative damage factors related to bone remodelling and bone formation [146].

## 4. Conclusions

This study confirms that added-value products can be prepared from food industry by-products combinations. However, it should be mentioned that ingredients quantities and their pre-treatment must be carefully selected. In this study, in most cases (except sea buckthorn), by increasing FBB content the beverages overall acceptability was increased, and the highest was obtained for the samples prepared with 5.0 and 7.5 g of blueberries FBB. A very strong positive correlation (*r* = 0.8525) between overall acceptability, evaluated by points, and emotion “happy”, induced for consumers by the prepared beverages, was found. Moreover, FBB is a good source to increase total phenolic compounds (TPC) content (in this study, on average, by 9.0%) in beverages. Finally, it can be stated that newly developed nutraceutical beverages are acceptable for consumers, induced positive emotions, as well as possessing desirable antimicrobial and antioxidant properties, and are prepared in an environmentally friendly and sustainable manner.

## Figures and Tables

**Table 1:** Parameters after 48 h of milk permeate fermented with LUHS29 strain.

Milk Permeate Samples	pH		TTA, No.		LAB Count, log_10_ CFU mL^−1^		Lactose, g 100 g^−1^		GOS mg 100 mL^−1^	
									G2	G3
MP_NF_	5.88 ± 0.80 ^b^		3.00 ± 0.14 ^a^		nd		10.48 ± 0.28 ^b^		nd	nd
MP_LUHS29_	3.91 ± 0.23 ^a^		9.50 ± 0.19 ^b^		8.19 ± 0.23		5.05 ± 0.19 ^a^		21.70 ± 0.33	5.10 ± 0.11
	**Overall Acceptability**	**Emotions Induced by the Beverages (from 0 to 1)**								
		**Neutral**	**Happy**	**Sad**	**Angry**	**Surprised**	**Scared**	**Disgusted**	**Contempt**	**Valence**
MP_NF_	5.20 ± 0.18	0.370 ± 0.020 ^b^	0.130 ± 0.003	0.180 ± 0.004 ^b^	0.060± 0.001 ^a^	0.03 ± 0.001 ^b^	0.0010 ± 0.00002	0.00100 ± 0.00002	0.0900 ± 0.0020 ^b^	0.080 ± 0.002 ^a^
MP_LUHS29_	5.30 ± 0.13	0.230 ± 0.004 ^a^	0.14 ± 0.003	0.160 ± 0.003 ^a^	0.130 ± 0.003 ^b^	0.010 ± 0.0002 ^a^	0.00100 ± 0.00002	0.00100 ± 0.00002	0.0300 ± 0.0006 ^a^	0.130 ± 0.003 ^b^

LAB, lactic acid bacteria; CFU, colony-forming units; TTA, total titratable acidity; G2, galactobiose; G3, galactotriose; MP, milk permeate; MP_LUHS29_, fermented with LUHS29 (*P. acidilactici);* MP_NF_, unfermented; GOS, galactooligosaccharides; nd, not determined. Data are represented as means (*n* = 3) ± SD. ^a–b^ Means with different letters in column are significantly different (*p* ≤ 0.05).

**Table 2:** The diameter of inhibition zones (mm) of the prepared beverages against pathogenic and opportunistic strains of milk permeate fermented with LUHS29 strain.

Samples	Diameter of Inhibition Zones (DIZ) (mm)														
	Pathogenic and Opportunistic Bacteria Strains														
	1	2	3	4	5	6	7	8	9	10	11	12	13	14	15
MP_LUHS29_	nd	nd	nd	nd	nd	nd	nd	nd	nd	12.7 ± 0.4	nd	nd	nd	nd	15.0 ± 0.1
MP_NF_	nd	nd	nd	nd	nd	nd	nd	nd	nd	nd	nd	nd	nd	nd	nd

MP, milk permeate; MP_LUHS29_, fermented with LUHS29 (P. acidilactici); MP_NF_, unfermented; nd, not determined; 1, Klebsiella pneumonia; 2, Salmonella enterica; 3, Pseudomonas aeruginosa; 4, Acinetobacter baumannii; 5, Proteus mirabilis, 6, MRSA M87fox; 7, Enterococcus faecalis; 8, Enterococcus faecium; 9, Bacillus cereus; 10, Streptococcus mutans; 11, Enterobacter cloacae; 12, Citrobacter freundii; 13, Streptococcus epidermis, 14, Staphylococcus haemolyticus; 15, Pasteurella multocida. Data are represented as means (*n* = 3) ± SD.

**Table 3:** Acidity (pH, total titratable acidity (TTA), and lactic acid isomers concentration) and microbiological parameters (lactic acid bacteria (LAB), mould/yeast (M/Y), total bacteria (TBC), and total enterobacteria (TEC) count) of the extruded and fermented wheat bran.

Wheat by-Product Samples	pH	TTA, No.	Lactic Acid Content, g 100 g^−1^		LAB	M/Y	TBC		TEC	
					log_10_CFU g^−1^					
	Duration of Fermentation, 24 h		L(+)	D(−)						
W_nonF_	6.04 ± 0.01 ^c^	0.10 ± 0.02 ^a^	-	-	5.20 ± 0.12 ^a^	4.26 ± 0.11	9.04 ± 0.14 ^b^		5.69 ± 0.23 ^b^	
W_ex130/screwspeed25_	5.91 ± 0.02 ^b^	0.20 ± 0.03 ^b^	-	-	5.34 ± 0.09 ^a^	4.38 ± 0.19	8.46 ± 0.10 ^a^		4.32 ± 0.14 ^a^	
W_ex130/screwspeed25_Lu	4.20 ± 0.01 ^a^	3.50 ± 0.10 ^c^	0.275 ± 0.013	0.203 ± 0.007	8.79 ± 0.12 ^b^	4.32 ± 0.07	8.84 ± 0.13 ^b^		nd	
	**Fructose**		**Glucose**		**Sucrose**			**Maltose**		
	**g 100 g^−1^**									
W_ex130/screwspeed25_	0.11 ± 0.02		nd		0.81 ± 0.07			0.11 ± 0.01		
W_ex130/screwspeed25_Lu	nd		nd		nd			nd		

W, wheat bran; _nonF_, non-fermented; Lu, fermented with *Lactobacillus uvarum*; _ex130/screwspeed25_—extruded at 130 °C, screw speed 25 rpm; TTA, total titratable acidity; LAB, lactic acid bacteria; M/Y, mould and yeast; TBC, total bacteria count; TEC, total enterobacteria count; CFU, colony-forming units; nd, not determined, not analysed. The data expressed as mean values (*n* = 3) ± SD; SD, standard deviation. ^a–c^ The mean values within a column with different letters are significantly different (*p* ≤ 0.05).

**Table 4:** The amino acids (g 100 g^−1^) and biogenic amines (mg kg^−1^) concentration in extruded and nontreated cereal by-products non-fermented and fermented with *L. uvarum* strain.

		W_nonF_	W_ex130/_ _screwspeed25_	W_ex130/_ _screwspeed25_Lu
**The** **amino** **acids,** **g 100g^−1^**	Asp	0.43 ± 0.03 ^a^	0.44 ± 0.03 ^a^	0.48 ± 0.04 ^a^
	Glu	1.75 ± 0.09 ^b^	1.43 ± 0.09 ^a^	1.47 ± 0.08 ^a^
	Asn	nd	nd	nd
	Ser	0.29 ± 0.03 ^a^	0.26 ± 0.02 ^a^	0.26 ± 0.02 ^a^
	His	0.12 ± 0.01 ^b^	0.10 ± 0.01 ^a^	0.11 ± 0.01 ^a^
	Gly	0.27 ± 0.02 ^a^	0.24 ± 0.02 ^a^	0.26 ± 0.02 ^a^
	Thr	0.25 ± 0.02 ^a^	0.25 ± 0.02 ^a^	0.26 ± 0.02 ^a^
	Arg	0.31 ± 0.03 ^b^	0.27 ± 0.02 ^a^	0.27 ± 0.02 ^a^
	Ala	0.24 ± 0.02 ^a^	0.21 ± 0.02 ^a^	0.23 ± 0.02 ^a^
	Tyr	0.18 ± 0.01 ^a^	0.19 ± 0.01 ^a^	0.17 ± 0.01 ^a^
	Cys	0.34 ± 0.03 ^a^	0.38 ± 0.03 ^b^	0.40 ± 0.03 ^b^
	Val	0.34 ± 0.03 ^a^	0.32 ± 0.03 ^a^	0.34 ± 0.03 ^a^
	Met	0.12 ± 0.01 ^a^	0.13 ± 0.01 ^a^	0.13 ± 0.01 ^a^
	Trp	0.36 ± 0.03 ^c^	0.32 ± 0.03 ^b^	0.29 ± 0.02 ^a^
	Phe	0.28 ± 0.02 ^b^	0.24 ± 0.02 ^a^	0.22 ± 0.02 ^a^
	Ile	0.40 ± 0.04 ^b^	0.32 ± 0.03 ^a^	0.32 ± 0.03 ^a^
	Leu	0.14 ± 0.01 ^b^	0.10 ± 0.01 ^a^	0.11 ± 0.01 ^a^
	Lys	0.26 ± 0.02 ^a^	0.29 ± 0.02 ^a^	0.34 ± 0.03 ^b^
	Pro	0.50 ± 0.04 ^c^	0.27 ± 0.02 ^b^	0.24 ± 0.02 ^a^
**BAs con-** **centration,** **mg kg^−1^**	PHE	nd	nd	nd
	PUT	102.3 ± 2.6 ^a^	154.1 ± 5.4 ^b^	160.1 ± 4.0 ^c^
	CAD	41.3 ± 1.6	nd	nd
	HIS	63.6 ± 2.2	nd	nd
	TYR	nd	nd	nd
	SPRMD	nd	nd	nd
	SPRM	111.9 ± 3.9 ^c^	32.50 ± 0.8 ^a^	33.6 ± 1.2 ^b^

W, wheat bran; _nonF_, non-fermented; Lu, fermented with *Lactobacillus uvarum*; _ex130/screwspeed25_, extruded at 130 °C, screw speed 25 rpm; nd, not determined; Asp, aspartic acid, Ala, alanine, Gly, glycine, Val, valine, Leu, leucine, Ile, isoleucine, Thr, threonine, Ser, serine, Pro, proline Asn, asparagine, Met, methionine, Glu, glutamine, Phe, phenylalanine, Lys, lysine, His, histidine, Arg, arginine, Tyr, tyrosine, Trp, tryptophan, Cys, cysteine; Bas, biogenic amines; PHE, phenylethylamine; PUT, putrescine; CAD, cadaverine; HIS, histamine; TYR, tyramine; SPRMD, spermidine; SPRM, spermine. Data are represented as means (*n* = 3) ± SD. ^a–c^, mean values within a row denoted with different letters are significantly different (*p* ≤ 0.05). nd, not determined.

**Table 5:** Antimicrobial properties of the fruit/berry by-products.

Samples	The Diameter of Inhibition Zones (DIZ) (mm)									
	Pathogenic and Opportunistic Bacterial Strains									
	1	2	3	4	5	6	7	8	9	10
Shepherd	n.d	13.3 ± 0.4 ^b^	n.d	n.d	n.d	n.d	n.d	12.9 ± 0.4 ^a^	n.d	n.d
Raspberries	n.d	n.d	12.0 ± 0.4	15.5 ± 0.3 ^b^	14.4 ± 0.3	12.2 ± 0.2	10.5 ± 0.4	13.6 ± 0.3 ^b^	n.d	15.3 ± 0.1 ^c^
Sea buckthorn	n.d	n.d	n.d	14.3 ± 0.4 ^a^	n.d	n.d	n.d	15.4 ± 0.2 ^c^	n.d	13.4 ± 0.2 ^b^
Blueberries	n.d	9.2 ± 0.2 ^a^	n.d	14.2 ± 0.1 ^a^	n.d	n.d	10.7 ± 0.21	12.4 ± 0.3 ^a^	n.d	12.3 ± 0.4 ^a^
**Experiment Design**										
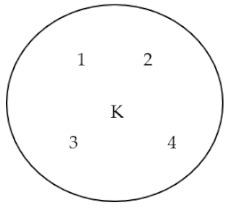		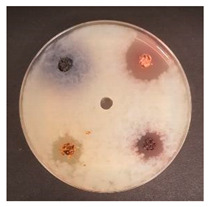			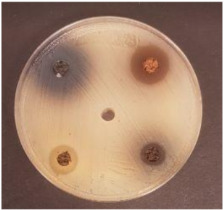			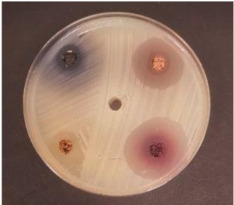		
Shepherd by-products; Raspberries by-products; Sea buckthorn by-products; Blueberry by-products; K–control.		*Bacillus pseudomycoides* LT 104			*Acinetobacter johnsonii* LT 110			*Cronobacter sakazakii* LT 106		

1—Salmonella enterica Infantis LT 101; 2—Staphylococcus aureus LT 102; 3, E. coli (hemolytic) LT 103; 4—Bacillus pseudomycoides LT 104; 5—Aeromonas veronii LT 105; 6—Cronobacter sakazakii LT 106; 7—Hafnia alvei LT 107; 8—Enterococcus durans LT 108; 9—Kluyvera cryocrescens LT 109; 10—Acinetobacter johnsonii LT 110. The data expressed as mean values (*n* = 3) ± SD; SD, standard deviation. ^a–c^ The mean values within a column with different letters are significantly different (*p* ≤ 0.05); n.d, not determined.

**Table 6:** Overall acceptability and emotions induced for consumers by the prepared beverages enriched with extruded and fermented wheat bran.

	Beverages Samples					
	MP_NF_	MP_F_	MP_LUHS29+2.5WB_	MP_LUHS29+5WB_	MP_LUHS29+7.5WB_	MP_LUHS29+10WB_
	Overall Acceptability					
	5.2 ± 0.2 ^b^	5.3 ± 0.1 ^b^	4.2 ± 0.1 ^a^	6.4 ± 0.1 ^d^	6.2 ± 0.2 ^c^	7.9 ± 0.2 ^e^
	Emotions Induced by the Beverages (from 0 to 1)					
Neutral	0.37 ± 0.02 ^c^	0.230 ± 0.004 ^a^	0.470 ± 0.013 ^d^	0.31 ± 0.01 ^b^	0.46 ± 0.01 ^d^	0.49 ± 0.01 ^e^
Happy	0.130 ± 0.003 ^c^	0.140 ± 0.003 ^d^	0.060 ± 0.002 ^b^	0.060 ± 0.002 ^b^	0.01000 ± 0.0002 ^a^	0.150 ± 0.004 ^e^
Sad	0.180 ± 0.004 ^e^	0.160 ± 0.003 ^d^	0.070 ± 0.002 ^c^	0.040 ± 0.001 ^a^	0.060 ± 0.001 ^b^	0.090 ± 0.003 ^c^
Angry	0.060 ± 0.001 ^d^	0.130 ± 0.003 ^f^	0.030 ± 0.001 ^c^	0.040 ± 0.001 ^b^	0.100 ± 0.002 ^e^	0.020 ± 0.001 ^a^
Surprised	0.03 ± 0.001 ^d^	0.0100 ± 0.0002 ^c^	0.0030 ± 0.0001 ^b^	0.080 ± 0.003 ^e^	0.00100 ± 0.00002 ^a^	0.00100 ± 0.00003 ^a^
Scared	0.00100 ± 0.00002 ^a^	0.00100 ± 0.00002 ^a^	0.020 ± 0.001 ^c^	0.0100 ± 0.0003 ^b^	0.00100 ± 0.00002 ^a^	0.00100 ± 0.00003 ^a^
Disgusted	0.00100 ± 0.00002 ^a^	0.00100 ± 0.00002 ^a^	0.0020 ± 0.0001 ^b^	0.00100 ± 0.00003 ^a^	0.00100 ± 0.00002 ^a^	0.0080 ± 0.0002 ^c^
Contempt	0.090 ± 0.002 ^d^	0.0300 ± 0.0006 ^b^	0.050 ± 0.001 ^c^	0.0100 ± 0.0003 ^a^	0.140 ± 0.003 ^e^	0.050 ± 0.001 ^c^
Valence	0.080 ± 0.002 ^c^	0.130 ± 0.003 ^f^	0.090 ± 0.002 ^d^	0.070 ± 0.002 ^b^	0.0300 ± 0.0007 ^a^	0.110 ± 0.003 ^e^

MP_NF_, non-fermented milk permeate; MP_F_, milk permeate fermented with LUHS29 (*P. acidilactici)*; WB, wheat bran extruded at 130 °C, screw speed 25 rpm and fermented with LUHS245 *(L. uvarum)*; 2.5, 5, 7.5, and 10, quantity of WB used, g 50 mL^−1^. Data are represented as means (*n* = 3) ± SD. ^a–f^ Means with different letters in column are significantly different (*p* ≤ 0.05).

**Table 7:** Overall acceptability and emotions induced for consumers by the prepared beverages enriched with extruded and fermented wheat bran and fruit/berry by-products.

Beverage Samples	Overall Acceptability	Emotions Induced by the Beverages (from 0 to 1)								
		Neutral	Happy	Sad	Angry	Surprised	Scared	Disgusted	Contempt	Valence
MP+10WB	7.9 ± 0.21 ^e^	0.49 ± 0.01 ^i^	0.150 ± 0.004 ^c^	0.090 ± 0.002 ^b^	0.0200 ± 0.0005 ^a^	0.00100 ± 0.00003 ^a^	0.00100 ± 0.00003 ^a^	0.0080 ± 0.0002 ^d^	0.050 ± 0.001 ^b^	0.090 ± 0.002 ^b^
2.5.ShepMP_LUHS29+10WB_	3.2 ± 0.11 ^b^	0.44 ± 0.02 ^h^	0.080 ± 0.003 ^a^	0.170 ± 0.006 ^c^	0.070 ± 0.002 ^b^	0.0090 ± 0.0003 ^d^	0.00100 ± 0.00003 ^a^	0.0020 ± 0.0001 ^b^	0.110 ± 0.004 ^e^	0.130 ± 0.004 ^c^
5.0.ShepMP_LUHS29+10WB_	7.6 ± 0.15 ^e^	0.21 ± 0.01 ^b^	0.190 ± 0.005 ^d^	0.260 ± 0.007 ^d^	0.070 ± 0.002 ^b^	0.033 ± 0.001 ^f^	0.00100 ± 0.00003 ^a^	0.0030 ± 0.0001 ^c^	0.100 ± 0.003 ^e^	0.090 ± 0.002 ^b^
7.5.ShepMP_LUHS29+10WB_	8.0 ± 0.14 ^f^	0.19 ± 0.00 ^a^	0.34 ± 0.01 ^f^	0.100 ± 0.002 ^b^	0.110 ± 0.003 ^c^	0.0050 ± 0.0001 ^c^	0.00100 ± 0.00002 ^a^	0.00100 ± 0.00002 ^a^	0.050 ± 0.001 ^b^	0.190 ± 0.004 ^b^
2.5. RaspMP_LUHS29+10WB_	2.6 ± 0.1 ^a^	0.180 ± 0.006 ^a^	0.050 ± 0.002 ^a^	0.37 ± 0.01 ^f^	0.170 ± 0.005 ^d^	0.0020 ± 0.0001 ^b^	0.00100 ± 0.00003 ^a^	0.039 ± 0.001 ^c^	0.35 ± 0.01 ^i^	0.180 ± 0.006 ^d^
5.0. RaspMP_LUHS29+10WB_	7.3 ± 0.1 ^d^	0.40 ± 0.01 ^g^	0.180 ± 0.005 ^d^	0.24 ± 0.01 ^d^	0.020 ± 0.001 ^a^	0.0020 ± 0.0001 ^b^	0.00100 ± 0.00003 ^a^	0.00100 ± 0.00003 ^a^	0.060 ± 0.002 ^c^	0.090 ± 0.002 ^b^
7.5 RaspMP_LUHS29+10WB_	8.6 ± 0.2 ^g^	0.32 ± 0.01 ^e^	0.360 ± 0.003 ^f^	0.39 ± 0.01 ^f^	0.020 ± 0.0004 ^a^	0.0090 ± 0.0002 ^d^	0.00100 ± 0.00002 ^a^	0.00100 ± 0.00002 ^a^	0.25 ± 0.01 ^g^	0.320 ± 0.01 ^f^
2.5. SeaMP_LUHS29+10WB_	5.6 ± 0.2 ^c^	0.47 ± 0.01 ^h^	0.13 ± 0.00 ^b^	0.25 ± 0.01 ^d^	0.13 ± 0.004 ^c^	0.0080 ± 0.0002 ^d^	0.001 ± 0.00003 ^a^	0.001 ± 0.00003 ^a^	0.22 ± 0.01 ^f^	0.47 ± 0.01 ^g^
5.0 SeaMP_LUHS29+10WB_	8.7 ± 0.2 ^h^	0.32 ± 0.01 ^e^	0.35 ± 0.00 ^f^	0.40 ± 0.01 ^f^	0.070 ± 0.002 ^b^	0.00100 ± 0.00003 ^a^	0.00100 ± 0.00003 ^a^	0.00100 ± 0.00003 ^a^	0.28 ± 0.01 ^h^	0.32 ± 0.01 ^f^
7.5 SeaMP_LUHS29+10WB_	7.7 ± 0.2 ^e^	0.26 ± 0.01 ^c^	0.25 ± 0.01 ^e^	0.30 ± 0.01 ^e^	0.060 ± 0.002 ^b^	0.0020 ± 0.0001 ^b^	0.00100 ± 0.00003 ^a^	0.00080 ± 0.00002 ^d^	0.050 ± 0.001 ^b^	0.260 ± 0.007 ^e^
2.5 BluMP_LUHS29+10WB_	7.3 ± 0.2 ^d^	0.38 ± 0.01 ^f^	0.190 ± 0.003 ^d^	0.170 ± 0.005 ^c^	0.0100 ± 0.0003 ^a^	0.0150 ± 0.0004 ^e^	0.00100 ± 0.00003 ^a^	0.00100 ± 0.00002 ^a^	0.080 ± 0.002 ^d^	0.060 ± 0.002 ^a^
5.0 BluMP_LUHS29+10WB_	9.3 ± 0.17 ^i^	0.28 ± 0.01 ^d^	0.360 ± 0.003 ^f^	0.190 ± 0.004 ^c^	0.0100 ± 0.0002 ^a^	0.00100 ± 0.00002 ^a^	0.00100 ± 0.00002 ^a^	0.00200 ± 0.00004 ^b^	0.030 ± 0.001 ^a^	0.28 ± 0.01 ^e^
7.5 BluMP_LUHS29+10WB_	9.6 ± 0.26 ^i^	0.27 ± 0.01 ^c^	0.480 ± 0.005 ^g^	0.070 ± 0.002 ^a^	0.0100 ± 0.0003 ^a^	0.0340 ± 0.001 ^f^	0.00100 ± 0.00003 ^a^	0.0030 ± 0.0001 ^c^	0.110 ± 0.003 ^f^	0.060±0.002 ^a^

2.5, 5.0, 7.5, quantities of fruit/berry by-products used, g 50 mL^−1^; MP_NF_, non-fermented milk permeate; MP_F_, milk permeate fermented with LUHS29 (*P. acidilactici)*; Shep, Shepherd/*Sambucus nigra, Rasp*, Raspberries/*Rubus idaeus; Sea*, Sea buckthorn/*Hippophae rhamnoides; Blu*, blueberries/*Vaccinium myrtillus;* WB, wheat bran extruded at 130 °C, screw speed 25 rpm and fermented with LUHS245 *(L. uvarum)*. 10WB, quantity of WB used, g 50 mL^−1^. Data are represented as means (*n* = 3) ± SD. ^a–i^ Means with different letters in column are significantly different (*p* ≤ 0.05).

**Table 8:** The diameter of inhibition zones (mm) of the prepared beverages against pathogenic and opportunistic strains.

Samples	The Diameter of Inhibition Zones (DIZ) (mm)									
	Pathogenic and Opportunistic Bacterial Strains									
	1	2	3	4	5	6	7	8	9	10
MP_LUHS29__+10WB_	9.1 ± 0.2 ^a^	nd	nd	nd	10.1 ± 0.4 ^b^	nd	nd	nd	nd	nd
MP_LUHS29__+10WB_+Shep7.5	10.2 ± 0.3 ^b^	10.3 ± 0.4 ^a^	13.4 ± 0.7 ^c^	10.3 ± 0.4 ^a^	9.0 ± 0.3 ^a^	9.3 ± 0.1 ^a^	9.2 ± 0.2 ^a^	12.3 ± 0.3 ^a^	nd	10.3 ± 0.4 ^a^
MP_LUHS29__+10WB_+Rasp7.5	13.0 ± 0.2 ^d^	10.1 ± 0.5 ^a^	9.3 ± 0.3 ^a^	13.6 ± 0.4 ^c^	12.3 ± 0.6 ^c^	9.6 ± 0.1 ^a^	nd	13.4 ± 0.2 ^b^	nd	14.3 ± 0.6 ^d^
MP_LUHS29__+10WB_+Sea5.0	10.3 ± 0.4 ^b^	11.0 ± 0.6 ^a;b^	10.2 ± 0.4 ^b^	10.3 ± 0.3 ^a^	12.4 ± 0.3 ^c^	10.4 ± 0.2 ^a;b^	nd	14.5 ± 0.4 ^c^	nd	13.2 ± 0.3 ^c^
MP_LUHS29__+10WB_+Blu7.5	12.4 ± 0.3 ^c^	12.6 ± 0.2 ^c^	14.0 ± 0.3 ^c^	12.1 ± 0.6 ^b^	12.2 ± 0.2 ^c^	12.3 ± 0.1 ^b^	10.3 ± 0.2 ^b^	15.6 ± 0.5 ^d^	nd	12.1 ± 0.4 ^b^

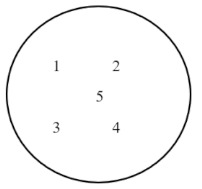		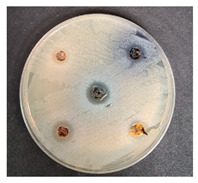			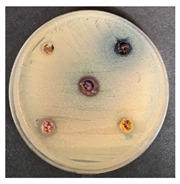			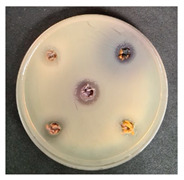		
MP_LUHS29+10WB_ MP_LUHS29+10WB_+Shep7.5 MP_LUHS29+10WB_+Rasp7.5 MP_LUHS29+10WB_+Sea5 MP_LUHS29+10WB_+Blu7.5		*Salmonella enterica Infantis* LT 101			*Staphylococcus aureus* LT 102			*E. coli* (hemolytic) LT 103		
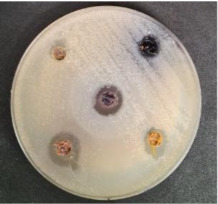		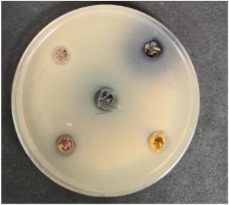			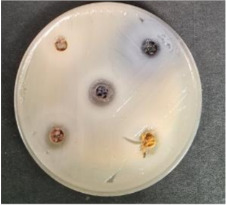			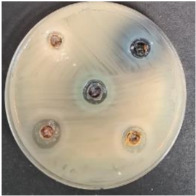		
*Bacillus pseudomycoi*desLT 104		*Aeromonas veronii*LT 105			*Cronobacter sakazakii* LT 106			*Acinetobacter johnsonii* LT 110		

1, Salmonella enterica Infantis LT 101; 2, Staphylococcus aureus LT 102; 3, E. coli (hemolytic) LT 103; 4, Bacillus pseudomycoides LT 104; 5, Aeromonas veronii LT 105; 6, Cronobacter sakazakii LT 106; 7, Hafnia alvei LT 107; 8, Enterococcus durans LT 108; 9, Kluyvera cryocrescens LT 109; 10, Acinetobacter johnsonii LT 110; nd, not determined; MP_NF_, non-fermented milk permeate; MP_F_, milk permeate fermented with LUHS29 (*P. acidilactici)*; Shep, Shepherd/*Sambucus nigra, Rasp*, Raspberries/*Rubus idaeus; Sea*, Sea buckthorns/*Hippophae rhamnoides; Blu*, blueberries/*Vaccinium myrtillus;* WB, wheat bran extruded at 130 °C, screw speed 25 rpm and fermented with LUHS245 *(L. uvarum)*; 10 WB, quantity of WB used, g 50 mL^−1^; 5.0, 7.5, quantity of berries used, g 50 mL^−1^. Data are represented as means (n = 3) ± SD. ^a–d^ Means with different letters in column are significantly different (*p* ≤ 0.05); nd, not determined.

**Table 9:** Viable lactic acid bacteria (LAB) count in prepared beverages during the four weeks of storage storage at +4 °C temperature.

Beverages Samples	LAB Count, log_10_ CFU mL^−1^				
	24 h	1st Week	2nd Week	3rd Week	4th Week
MP_NF_	n.d	n.d	n.d	n.d	n.d
MP_F_	7.99 ± 0.22 ^a^	7.89 ± 0.16 ^a^	7.79 ± 0.19 ^b^	6.99 ± 0.24 ^a^	5.80 ± 0.17 ^a^
MP_LUHS29__+10WB_	8.20 ± 0.18 ^c^	8.01 ± 0.20 ^b^	8.00 ± 0.28 ^b^	8.01 ± 0.20 ^b^	7.20 ± 0.21 ^b^
MP_LUHS29__+10WB_+Shep7.5	8.33 ± 0.21 ^d^	8.03 ± 0.15 ^b^	7.83 ± 0.27 ^b^	7.80 ± 0.27 ^b^	6.93 ± 0.20 ^b^
MP_LUHS29__+10WB_+Rasp7.5	8.17 ± 0.26 ^b^	7.98 ± 0.19 ^b^	7.91 ± 0.20 ^b^	7.69 ± 0.19 ^b^	5.88 ± 0.14 ^a^
MP_LUHS29__+10WB_+Sea5.0	8.10 ± 0.12 ^b^	7.99 ± 0.16 ^b^	7.90 ± 0.27 ^b^	7.54 ± 0.25 ^b^	5.67 ± 0.13 ^a^
MP_LUHS29__+10WB_+Blu7.5	8.15 ± 0.21 ^b^	7.87 ± 0.29 ^a^	7.31 ± 0.28 ^a^	6.99 ± 0.27 ^a^	5.94 ± 0.16 ^a^

LAB, lactic acid bacteria; MP_NF,_ non-fermented milk permeate; MP_F_, milk permeate fermented with LUHS29 (*P. acidilactici)*; Shep, Shepherd/*Sambucus nigra, Rasp*, Raspberries/*Rubus idaeus*; *Sea*, Sea buckthorns/*Hippophae rhamnoides; Blu—*blueberries/*Vaccinium myrtillus;* WB, wheat bran extruded at 130 °C, screw speed 25 rpm and fermented with LUHS245 *(L. uvarum)*; 10 WB, quantity of WB used, g 50 mL^−1^; 5.0, 7.5, quantity of berries used, g 50 mL^−1^. The data expressed as mean values (*n* = 3) ± SD; SD, standard deviation. ^a–d^ The mean values within a column with different letters are significantly different (*p* ≤ 0.05); n.d, not determined.

**Table 10:** Colour coordinates, acidity and antioxidant parameters of the prepared beverages.

BeveragesSamples	Colour Coordinates, NBS			pH	TTA, °N	TPC, mg 100 g^−1^ d.m.	Antioxidant Activity, %
	L*	a*	b*				
MP_NF_	27.3 ± 1.7 ^b^	2.01 ± 0.06 ^b^	1.11 ± 0.03 ^b^	5.88 ± 0.2 ^f^	3.0 ± 0.1 ^a^	68.2 ± 3.7 ^a^	14.1 ± 1.3 ^a^
MP_F_	31.4 ± 2.9 ^c^	1.83 ± 0.05 ^a^	1.34 ± 0.04 ^c^	3.91 ± 0.02 ^a^	9.5 ± 0.2 ^d^	104.8 ± 5.9 ^b^	21.7 ± 1.6 ^b^
MP_LUHS29+10WB_	39.1 ± 2.3 ^f^	1.77 ± 0.06 ^a^	1.92 ± 0.06 ^d^	4.30 ± 0.01 ^d^	8.5 ± 0.2 ^c^	124.4 ± 4.1 ^c^	25.8 ± 1.8 ^c^
MP_LUHS29__+10WB_+Shep7.5	19.7 ± 1.6 ^a^	5.31 ± 0.17 ^c^	0.84 ± 0.03 ^a^	4.26 ± 0.03 ^d^	8.8 ± 0.2 ^c^	132.5 ± 6.2 ^d^	29.3 ± 1.9 ^d^
MP_LUHS29__+10WB_+Rasp7.5	27.2 ± 1.8 ^b^	15.6 ± 0.8 ^e^	5.97 ± 0.26 ^e^	4.17 ± 0.02 ^b^	9.0 ± 0.3 ^c^	141.7 ± 7.1 ^e^	29.3 ± 1.7 ^d^
MP_LUHS29__+10WB_+Sea5.0	30.4 ± 2.9 ^c^	5.29 ± 0.23 ^c^	15.5 ± 1.4 ^f^	4.62 ± 0.02 ^e^	7.9 ± 0.3 ^b^	125.9 ± 4.5 ^c^	26.1 ± 2.0 ^c^
MP_LUHS29__+10WB_+Blu7.5	20.4 ± 1.6 ^a^	6.36 ± 0.17 ^d^	0.82 ± 0.02 ^a^	4.20 ± 0.01 ^c^	8.9 ± 0.2 ^c^	132.8 ± 4.6 ^d^	27.5 ± 1.6 ^d^

L*, lightness; a*, redness (a* greenness); b*, yellowness (b* blueness); TTA, total titratable acidity; LAB, lactic acid bacteria; TPC, total phenolic compounds. MP_NF,_ non-fermented milk permeate; MP_F_, milk permeate fermented with LUHS29 (*P. acidilactici)*; Shep, Shepherd/*Sambucus nigra, Rasp*, Raspberries/*Rubus idaeus; Sea*, Sea buckthorn/*Hippophae rhamnoides; Blu*, blueberries/*Vaccinium myrtillus;* WB, wheat bran extruded at 130 °C, screw speed 25 rpm and fermented with LUHS245 *(L. uvarum)*; 10WB, quantity of WB used, g 50 mL^−1^; 5.0, 7.5, quantity of berries used, g 50 mL^−1^. The data expressed as mean values (*n* = 3) ± SD; SD, standard deviation. ^a–f^ The mean values within a column with different letters are significantly different (*p* ≤ 0.05).

**Table 11:** Correlation coefficients between colour coordinates and overall acceptability and emotions induced for consumers by the tested beverages.

Colour Coordinates	Overall Acceptability	Neutral	Happy	Sad	Angry	Surprised	Scared	Disgusted	Contempt	Valence
	Correlation Coefficients (R) between Colour Coordinates and Overall Acceptability and Emotions Induced for Consumers by the Tested Beverages.									
L*	–0.2472	0.7466	–0.6405	0.1461	–0.0861	–0.4778	0.2659	0.5806	–0.0196	0.0491
a*	0.5451	–0.1683	0.6144	0.5909	–0.4113	–0.0543	0.0369	–0.3109	0.6709	0.6530
b*	0.3512	0.0988	0.2708	0.8350	–0.0416	–0.4592	0.0483	–0.2360	0.8582	0.7929

L*, lightness; a*, redness (a* greenness); b*, yellowness (b* blueness).
